# Forest Microhabitat Affects Succession of Fungal Communities on Decomposing Fine Tree Roots

**DOI:** 10.3389/fmicb.2021.541583

**Published:** 2021-01-28

**Authors:** Petr Kohout, Radka Sudová, Vendula Brabcová, Stanislav Vosolsobě, Petr Baldrian, Jana Albrechtová

**Affiliations:** ^1^Institute of Microbiology of the Czech Academy of Sciences, Prague, Czechia; ^2^Institute of Botany of the Czech Academy of Sciences, Pruhonice, Czechia; ^3^Department of Experimental Plant Biology, Faculty of Science, Charles University, Prague, Czechia

**Keywords:** soil organic matter, root litter, forest ecosystem, dark septate endophytes, fungal communities, forest microhabitats, stem decapitation, Norway spruce

## Abstract

Belowground litter derived from tree roots has been shown as a principal source of soil organic matter in coniferous forests. Fate of tree root necromass depends on fungal communities developing on the decaying roots. Local environmental conditions which affect composition of tree root mycobiome may also influence fungal communities developing on decaying tree roots. Here, we assessed fungal communities associated with decaying roots of *Picea abies* decomposing in three microhabitats: soil with no vegetation, soil with ericoid shrubs cover, and *P. abies* deadwood, for a 2-year period. Forest microhabitat showed stronger effect on structuring fungal communities associated with decaying roots compared to living roots. Some ericoid mycorrhizal fungi showed higher relative abundance on decaying roots in soils under ericoid shrub cover, while saprotrophic fungi had higher relative abundance in roots decomposing inside deadwood. Regardless of the studied microhabitat, we observed decline of ectomycorrhizal fungi and increase of endophytic fungi during root decomposition. Interestingly, we found substantially more fungal taxa with unknown ecology in late stages of root decomposition, indicating that highly decomposed roots may represent so far overlooked niche for soil fungi. Our study shows the importance of microhabitats on the fate of the decomposing spruce roots.

## Introduction

Soil organic matter (SOM) represents an important pool of carbon and nutrients. Globally, SOM contains more than three times as much carbon as either the atmosphere or terrestrial vegetation (Schmidt et al., 2011). SOM is created during accumulation and decomposition of wood and leaf litter on the soil surface and root litter within soil, as well as during translocation of currently photosynthesized organic compounds belowground by living plants (Litton et al., 2007). Large part of the belowground translocated carbon (C) enables to establish and maintain the root microbiome, which largely affects the physiology of the host plant. Belowground litter derived from roots and root-associated microorganisms has been shown as a principal source of SOM in ecosystems dominated by coniferous forests with understory vegetation dominated by ericoid dwarf shrubs (Clemmensen et al., 2013).

The tree root microbiome is comprised of a variety of mutualistic, endophytic, and parasitic microorganisms. Ectomycorrhizal fungi (EcMF) are recognized as one of the most important ecological groups. Although saprotrophic capabilities of the EcMF significantly decreased during their evolution (Kohler et al., 2015), some of them still produce enzymes, which might have the potential to decompose nutrient-rich and carbon-rich biopolymers, such as celluloses, hemicelluloses, lignin, etc. (Talbot et al., 2008). However, these lignocellulose-degrading capabilities are often considered to be rather limited (Baldrian, 2009) and vary widely across EcMF taxa (Bödeker et al., 2014; Kohler et al., 2015). Some EcMF take part in the oxidative decomposition of SOM such as the humic acids, which leads to mobilization of organic N and makes organic matter available for further degradation by other saprotrophic microorganisms (Lindahl and Tunlid, 2015; Shah et al., 2016).

Besides mycorrhizal fungi, tree roots harbor diverse communities of endophytic fungi (Jumpponen and Trappe, 1998). Compared to mycorrhizal fungi, the effect of fungal endophytes on host plant physiology is less clear and may range from positive to negative (Newsham, 2011; Reininger et al., 2012; Lukešová et al., 2015). Endophytic lifestyle is known among fungi from most of the fungal phyla. The endophytic communities associated with roots of trees in temperate and boreal regions are mainly dominated by the so-called dark septate endophytes (DSE) from *Phialocephala fortinii*–*Acephala applanata* complex (Grünig et al., 2008). Interestingly, the ability to endophytically colonize tree roots is also possessed by several ericoid mycorrhizal fungi (Chambers et al., 2008; Vohník et al., 2013), which primarily form mycorrhizal association with ericoid dwarf shrubs, such as *Vaccinium myrtillus*, commonly occurring on the forest floor (Smith and Read, 2008). Compared to EcMF, endophytic and ericoid mycorrhizal fungi (ErMF) generally feature greater enzymatic capabilities for degradation of complex organic compounds (Schlegel et al., 2016; Martino et al., 2018; Perotto et al., 2018).

Current research on decomposition of litter largely focuses on the aboveground component of plants and much less attention is paid to the root litter decomposition, partly because of the technical challenges (Langley et al., 2006; Li et al., 2015). However, decaying roots represent an important C source for microbial communities (Rasse et al., 2005) and play an important role in plant-soil feedback (Freschet et al., 2013). Because of a lower nutrient concentration and higher content of less degradable structural polymers, such as lignin, roots are considered to be more recalcitrant than aboveground litter and this might have an effect on their degradability by microorganisms (Jacobs et al., 2018).

Compared to decomposing leaf litter, where fungal communities undergo rapid succession with dramatic changes in the composition and often early decomposing taxa are completely replaced by late decomposers (Voříšková and Baldrian, 2013), fungal communities develop gradually on decomposing roots (Kohout et al., 2018; Otsing et al., 2018; Herzog et al., 2019). In fact, many fungal taxa, which are involved in tree root decomposition, are already present in living root tissue (Kohout et al., 2018). Therefore, composition of the fungal communities associated with living tree roots can subsequently affect succession of fungal communities on root necromass as well as root decomposition processes themselves. During early stages of the decomposition, a colonization priority effect can provide a competitive advantage to root endophytes or ErMF (Kohout et al., 2018), despite possessing lower copy numbers of genes encoding lignocellulose degrading enzymes than saprotrophic lignocellulose-decomposing fungi (Kohler et al., 2015; Schlegel et al., 2016; Martino et al., 2018).

It is widely acknowledged that a variety of environmental conditions, such as soil chemistry, climate, or host plant identity (Kolaříková et al., 2017; Põlme et al., 2018; van der Linde et al., 2018; Větrovský et al., 2019), can affect the composition of tree root-associated fungal communities. Forest microhabitats represent a natural source of such environmental variability in mature forest stands. Several studies documented that different tree root fungal communities are associated with different forest microhabitats (Iwański and Rudawska, 2007; Tedersoo et al., 2008). Decomposing tree trunks (termed coarse woody debris, CWD) may harbor less fungal pathogens and more wood decomposers compared to the forest floor soil (O’Hanlon-Manners and Kotanen, 2004). In addition, dead wood has higher capacity to retain moisture (DeLong et al., 1997) that may significantly affect germination and regeneration of tree seedlings (Veblen, 1989) as well as fungal communities associated with the roots of those trees that proliferate on CWD. Ericoid dwarf shrubs represent another factor that may affect the composition of fungal communities on tree roots (Kohout et al., 2011). Ericoid mycorrhizal fungi may act as endophytes in neighboring tree roots (Vohník et al., 2013; Sietiö et al., 2018). Due to the capability of ericoid mycorrhizal fungi to degrade recalcitrant compounds of plant cell walls (Vohník et al., 2012; Martino et al., 2018), decomposition of tree roots in ericoid shrubs can also differ from other forest microhabitats.

The main aim of our study was to compare the development of root-associated fungal communities during the decomposition of roots of *Picea abies* seedlings in three different forest microhabitats within a *P. abies*-dominated temperate forest: (i) bare forest soil, (ii) soil covered with ericoid shrubs (*Vaccinium myrtillus*), and (iii) coarse woody debris of *P. abies*. Considering that many fungal species that act in early phases of root decomposition possess endophytic or ericoid mycorrhizal lifestyle (Kohout et al., 2018), we hypothesized that the spruce roots decomposing in ericoid shrubs will harbor fungal communities enriched by ErMF. Besides that, we also expected that fungal communities associated with decomposing spruce roots in CWD microhabitats will contain a relatively higher amount of wood decomposing, saprotrophic fungi.

## Materials and Methods

### Study Site and Sampling Design

Study was conducted in a 1-ha, >100-years-old unmanaged spruce forest stand in the Bohemian Forest National Park, Czechia (longitude 49.0426N, latitude 13.618E; 1,190 m a.s.l.). Besides dominating spruce, only a few isolated *Fagus sylvatica* and *Sorbus aucuparia* trees occur in the study area. Forest floor was mostly covered with dense patches of shrubby vegetation comprised mostly by *Vaccinium myrtillus* and few bushes of *V. vitis-idaea*. Understory vegetation also included numerous naturally regenerating spruce seedling and several graminoid species (such as *Avenella flexuosa* and *Deschampsia cespitosa*). Besides the understory vegetation, the forest floor also included decomposing spruce logs. Naturally regenerating spruce seedlings were mostly observed on brown-rotten spruce logs of decay classes IV and V (based on Renvall, 1995). Therefore, forest floor was comprised of mosaic of different microhabitats, where spruce seedlings were regenerating.

To determine changes in fungal communities associated with decomposition processes of spruce roots in different forest microhabitats, we decided to study decomposing roots without disconnecting the root–soil interface. We believe that this practice will help us to determine naturally developing fungal communities associated with decomposing roots in the natural context, avoiding soil disturbance, and introduction of allochthonous material that would be required if root litter bags are used. In June 2015, more than 50 healthy-looking spruce seedlings of approximately same age (4–6 years old) were selected, in spatially random design, from (i) bare forest soil, (ii) soil covered by the ericoid shrubs of *Vaccinium myrtillus*, and (iii) CWD, approximately 150 seedlings in total. The seedlings were decapitated, i.e., cut off few centimeters aboveground, so that the remaining stem part remained visible and clearly marked. Roots of these decapitated spruce seedlings were subsequently sampled at four sampling times (2, 4, 10, and 15 months after the decapitation) to identify fungal communities developing on spruce roots. Besides the decapitated spruce seedlings, we also sampled “control” plants from the same microhabitats during all sampling times as well as in June 2015 during the time of experiment establishment. These control plants were selected based on the same criteria as the decapitated seedlings, also in spatially random design. Incorporation of the control plants into the study helped us to distinguish changes in fungal community composition during the spruce roots decomposition from shifts caused by tree aging and seasonal effects. At each sampling time, we collected eight decapitated and eight control spruce seedlings within each microhabitat which resulted in 48 samples per sampling time in total. Sampled seedlings were at least 5 m apart. Altogether, 216 samples were collected in this study. No samples were pooled. When sampled, decapitated and control seedlings were carefully pulled out of soil or decomposing wood. Only roots attached to spruce seedling stem were collected. Root samples were transported into lab within 3 h and stored in 5°C in plastic bags for up to 1 day until processed. Root systems were carefully washed under running tap water until no soil remained attached to them and then they were cut into 5 cm fragments. Ten fragments of fine roots (<2 mm diameter) per sample (approximately 0.25 g of fresh weight) were frozen and stored at −20°C for subsequent DNA isolation. These roots contained the ectomycorrhizal roots tips as well as the basal roots without the mycorrhizal colonization. Remaining fine roots were dried at 50°C and used for analyses of cellulose and lignin concentration.

### Molecular Analyses

All frozen root samples were disrupted in the tissue lyzer. Subsequently, DNA was extracted using the NucleoSpin PlantII Kit (Macherey-Nagel, Germany) according to the manufacturer’s instructions and eluted in 100 μl double distilled water (ddH_2_O). The fungal internal transcribed spacer 2 of the rDNA (ITS2) was amplified in triplicate to reduce PCR bias using fungal specific combination of primers fITS7 (Ihrmark et al., 2012) and ITS4 (White et al., 1990). Both primers were tagged with 10-base molecular identifiers (MID). Approximately 5 μl of DNA extract was used as template in PCR. The PCRs were conducted with Taq Pfu DNA Polymerase (Thermo Scientific) with final concentrations of 1x Pfu buffer, 2 mM MgSO_4_, 200 μM of each dNTP, and 0.5 μm of each primer in a total volume of 20 or 25 μl. The PCR conditions were as follows: initial denaturation at 95°C for 5 min, followed by 50 cycles with 95°C for 1 min, 61°C for 45 s, and 72°C for 2 min, concluded by a final elongation at 72°C for 10 min. PCR products were visualized on a 1% agarose gel. Successfully amplified samples were loaded on a 1% agarose gel and bands of the expected size were cut from the gel and processed with the Zymoclean Gel DNA recovery kit (Zymoclean). The DNA concentration in the eluates was measured using a Qubit 2.0 fluorometer with the HS kit and products were equimolarly pooled. Negative control with ddH_2_O instead of template DNA was produced with a specific identifier in the same way as the experimental samples and sequenced together with them. Genomic DNA of PhiX virus was used as a positive control for the Illumina MiSeq run. Sequencing was performed using 250-bp paired-end chemistry on an Illumina MiSeq system. The obtained data were deposited in NCBI SRA (PRJNA634919).

### Bioinformatical Analyses

SEED pipeline version 2.0 (Větrovský et al., 2018) was used for filtering and trimming the reads obtained from Illumina MiSeq. The reads were merged into paired end sequences with at least 30 bp overlap and maximum difference 10% (Aronesty, 2011). All sequences with ambiguous bases and average base quality scores lower than 38 were removed from the dataset. Sequences without primers and identifiers as well as sequences with mismatched identifiers were also removed. Remaining sequences were sorted into samples according to the MID sequences. Duplicates were removed prior to ITS2 extraction by ITSx (Bengtsson-Palme et al., 2013). The non-fungal ITS2 sequences were removed from the dataset and fungal ITS2 sequences were clustered using UPARSE (Edgar, 2013) implementation in VSEARCH version 1.11.2 on 98.5% similarity level. All global singletons, doubletons, and chimeric sequences were excluded. From each cluster, the most abundant sequence was selected as a representative sequence for subsequent analysis. BLAST search (Camacho et al., 2009) of the representative sequences was performed against local ITS database (UNITE repository, 08/22/2018 release). Representative sequences of fungal OTUs were assigned to closest species hypothesis (SH; Kõljalg et al., 2013) on 98.5% similarity level. The taxonomy of fungi follows Index Fungorum^1^.

The fungal OTUs were assigned to ecophysiological categories using FUNGuild (Nguyen et al., 2016) with subsequent manual corrections as follows: OTUs with sequence similarity to an INSD fungal sequence lower than 94% as well as assignments with confidence ranking “possible” were considered as unassigned. Besides that, we also corrected some ambiguous assignments based on our best knowledge. For example, *Meliniomyces variabilis* was considered as ericoid mycorrhizal, while other *Meliniomyces* spp. were treated as endophytic. Final fungal community matrix and taxonomy is provided in Supplementary Tables 1, 2.

### Determination of Root Tissue Chemistry

Part of the dried fine spruce roots was milled and subjected to determination of cellulose and lignin contents. Root lignin and cellulose contents were determined after milling and homogenizing dry root mass. Root lignin content was assessed from a 0.03 g root dry mass sample by thioglycolate solubilization (Lange et al., 1995) and then determined spectrophotometrically at a wavelength of 280 nm using hydrolytic lignin (Aldrich Chemical Company, United States) as a standard. Root cellulose content was analyzed based on Loader et al. (1997) from pellets after lignin extraction, which was repeatedly washed with 17% NaOH, dried and related to original dry weight, thus, the values are informative for comparison of different treatments.

### Statistical Analyses

All statistical analyses were conducted in R v. 3.2.3 (R Core Team, 2020). In most cases, differences at *P* < 0.05 were considered statistically significant. The only exception represented indicative species analysis, where differences at *P* < 0.01 were considered statistically significant. Differences in plant structural polymer (cellulose and lignin) concentrations among microhabitats and sampling times were tested using analysis of variance (ANOVA). The assumptions of ANOVA (homogeneity of variances and normal distribution) were tested prior to the analysis, and logarithmic transformation was applied when needed. *Post hoc* comparisons among means were conducted using Tukey HSD test.

Prior to subsequent analyses of fungal community composition, the number of sequences per sample was subsampled to median number of sequences per sample. Subsampling to the median rather than the minimum number appeared to improve beta diversity analysis since it retains higher resolution where possible (de Cárcer et al., 2011). Lowest number of sequences per sample was 4,959 in our study. Then, fungal OTU abundances were standardized using Hellinger transformation. Bray–Curtis dissimilarity was used to construct a fungal community dissimilarity matrix. To address the relative importance of microhabitat type and sampling time (time after seedling decapitation) and microhabitat × sampling time interaction on structure of the fungal communities and relative abundance of fungal ecophysiological categories associated with spruce seedling roots, we used a PERMANOVA as implemented in the adonis routine of the Vegan package of R (Oksanen et al., 2012). *Post hoc* test represented by pairwise PERMANOVA with 99,999 permutations was performed for fungal communities and fungal ecophysiological categories between sampling timepoints and between microhabitats to test the equality of group means (for sampling timepoints: 2, 4, 10, and 15 months and for microhabitats: forest bare soil, ericoid shrubs, and coarse woody debris). Bonferroni correction was used to calculate the corrected *P*-value determined by the pairwise PERMANOVA analysis. Indicator fungal OTUs for different microhabitat types and sampling times were determined using “indVal” function of the labdsv package of R (Roberts, 2015), where the significance of indicator values is determined based on a permutation test (only OTUs found in more than four samples were included in the analysis).

## Results

### Shifts in Cellulose and Lignin Concentrations in Decomposing Spruce Seedling Roots

**FIGURE 1:**
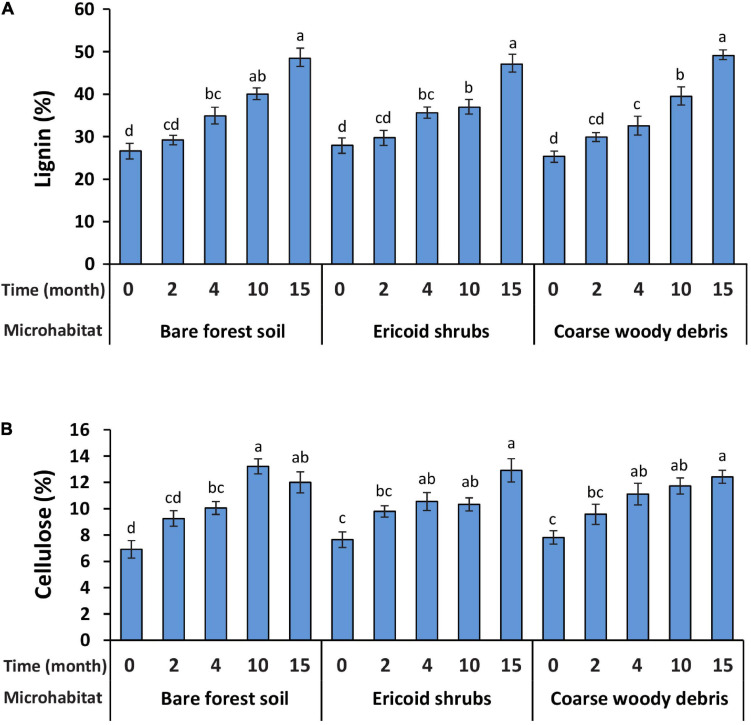
Concentrations of two plant structural biopolymers lignin **(A)** and cellulose **(B)** in root dry mass of *Picea abies* seedlings in the three studied microhabitats before (month 0) and after decapitation (months 2–15). Bars indicate means (*n* = 8) and whiskers represent standard errors. Different letters indicate significant differences among different sampling times within the same microhabitat. The values of cellulose are only indicative of relative differences among treatments, not corresponding to absolute values due to the method used.

Spruce seedling decapitation was associated with shifts in cellulose and lignin concentrations in root biomass. We observed significant increase in cellulose and lignin concentrations in decomposing spruce roots 4 months after the decapitation in all studied microhabitats. While cellulose concentration remained unchanged after the first significant increase, the lignin concentration in decomposing roots was gradually increasing during the whole experiment (Figure 1). Neither cellulose nor lignin concentration in root biomass was significantly affected by microhabitat type and microhabitat × time interaction. Consistent effect of sampling time on lignin and cellulose concentrations was not observed in control samples (Supplementary Figure 1).

### Shift in Fungal Ecophysiological Categories on Decomposing Spruce Seedling Roots

**FIGURE 2:**
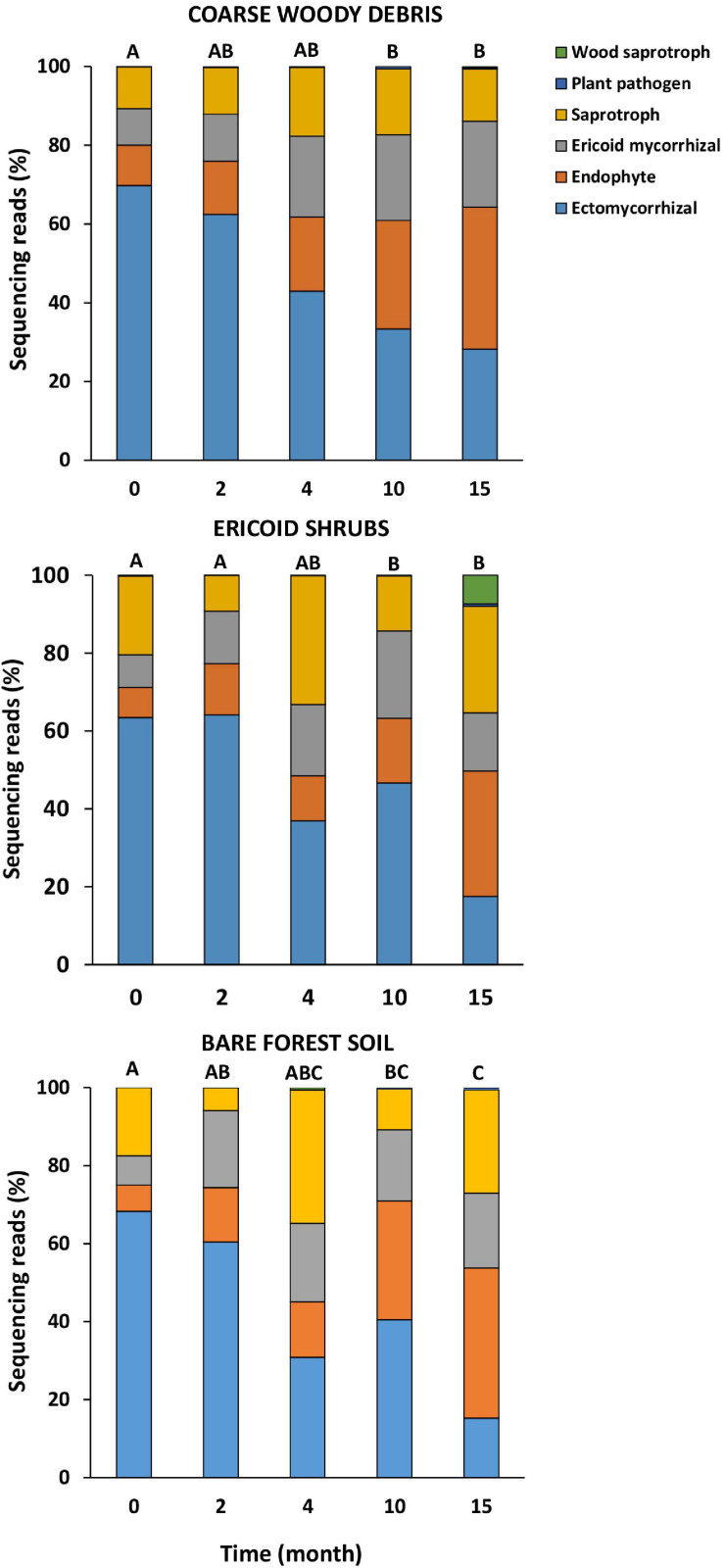
Relative numbers of sequences of major fungal ecophysiological categories on roots of *Picea abies* seedlings before (month 0) and after decapitation (month 2–15). Values represent means (*n* = 8) of the relative abundances of ITS2 amplicons expressed as percentages. Different letters indicate significant differences among sampling times within the same microhabitat.

The fungal communities associated with roots of living (control) *P. abies* seedlings were dominated by EcMF (62%), followed by saprotrophs (15%), ErMF (8%), and root endophytes (7%). Composition of fungal ecophysiological categories on roots of control plants was not significantly changing with time or among the studied microhabitats (Table 1). Therefore, we consider seasonality to have a minor effect on the composition of fungal ecophysiological categories of spruces seedlings on our study site. On the contrary, time after decapitation had strongly significant effect on relative abundance of fungal ecophysiological categories on spruce decaying roots (Table 1). The first significant difference in relative abundance of fungal ecophysiological categories between decaying roots and living roots was observed 10 months after the decapitation in all studied microhabitats (Figure 2). Subsequently, the proportions of fungal ecophysiological categories on decomposing roots changed gradually during succession. In general, we observed decline in relative abundance of EcMF, while root endophytes and ErMF tended to increase. Fifteen months after the decapitation, the decomposing roots of *P. abies* seedlings contained mostly sequences of endophytes (32%), followed by saprotrophs (20%), EcMF (18%), and ErMF (17%). Composition of fungal ecophysiological categories on decaying spruce roots was not significantly affected by microhabitat.

**TABLE 1:** Results of PERMANOVA analysis of sampling time and microhabitat and their interaction affecting relative abundance of fungal ecophysiological categories on decaying or living fine roots of *Picea abies* seedlings.

*Picea abies* roots	Tested variable	*R*^2^	*P*-value
Decaying roots (decapitated *P. abies* seedlings)	Sampling time	**0.227**	<0.001
	Microhabitat	n.d.	0.532
	Interaction	n.d.	0.487
Living roots (control)	Sampling time	n.d.	0.527
	Microhabitat	n.d.	0.822
	Interaction	n.d.	0.125

*Significant results are in a bold. *R*^2^ values were not determined (n.d.) for non-significant variables.*

### Succession of Fungal Communities on Decomposing Spruce Seedling Roots

**FIGURE 3:**
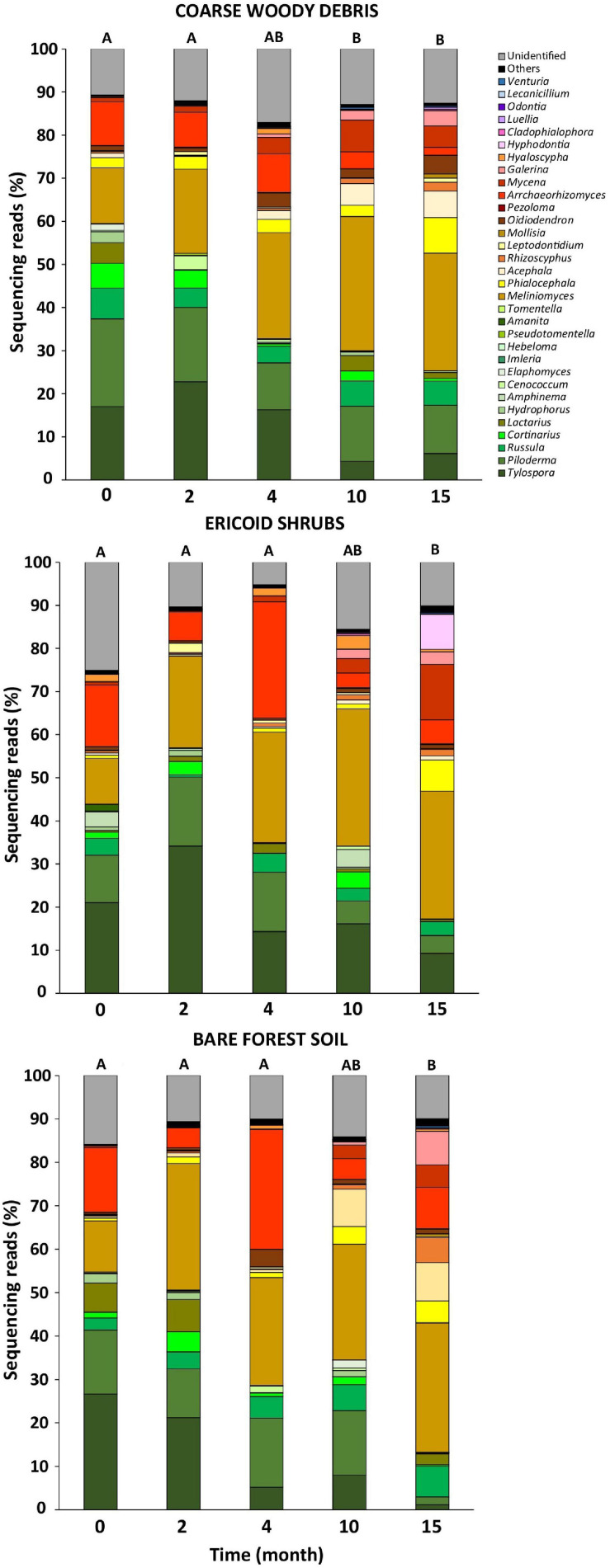
Relative abundance of major fungal genera on *Picea abies* seedling roots before (month 0) and after decapitation (month 2–15). Values represent means (*n* = 8) of the relative abundances of ITS2 amplicon reads expressed as percentages. Different letters indicate significant differences in fungal community composition among sampling times (A-B).

The fungal communities associated with roots of control *P. abies* seedlings were dominated by EcMF genera *Tylospora* (22% of fungal sequences), *Piloderma* (19%), and endophytic *Meliniomyces* spp. (12%). Surprisingly, we did not observe any effect of time on composition of fungal communities associated with control plants what indicates a negligible seasonality of fungal communities at our study site (Table 2). Therefore, we assume that all shifts in composition of fungal communities on decomposing spruce roots are associated with seedlings decapitation. Indeed, the time after decapitation structured fungal communities developing on the decomposing spruce roots (Table 2). The significant shift in fungal community composition was observed 10–15 months after the spruce seedlings decapitation (Figure 3). Indicative species analysis identified several OTUs of ErMF more frequently occurring on spruce roots after the spruce seedlings decapitation. Interestingly, we also observed a positive relationship between time after decapitation and number of indicative fungal taxa with endophytic and unknown ecologies (Table 3). OTUs with high affinity to the Ascomycota phylum prevailed among the indicative fungi with unknown ecology.

**TABLE 2:** Results of PERMANOVA analysis of sampling time and microhabitat and their interaction affecting composition of fungal communities associated with decaying or living fine roots of *Picea abies* in the studied microhabitats.

*Picea abies* roots	Tested variable	*R*^2^	*P*-value
Decaying roots (decapitated *P. abies* seedlings)	Sampling time	**0.086**	<0.001
	Microhabitat	**0.045**	<0.001
	Interaction	n.d.	<0.103
Living roots (control)	Sampling time	n.d.	0.117
	Microhabitat	**0.022**	0.014
	Interaction	n.d.	0.585

*Significant results are in a bold. *R*^2^ values were not determined (n.d.) for non-significant variables.*

**TABLE 3:** Significant relations between sampling time (month 0 refers to the control sampling before decapitation) and the occurrence of fungal OTUs associated with decapitated spruce roots, with classification into fungal species hypothesis on 98.5% similarity level.

	Species hypothesis	Taxonomy	Ecology^1^	Indicative for sampling time (month)^2^				
				0	2	4	10	15
OTU768	SH190469.07FU	*Russula*	EcM		*	*	*	*
OTU200	SH212614.07FU	*Hygrophorus*	EcM	*	*	*	*	
OTU2079	SH196824.07FU	*Piloderma*	EcM	*				
OTU113	SH188507.07FU	*Cortinarius*	EcM		*			
OTU74	SH213927.07FU	*Tylospora*	EcM	*	*	*	*	
OTU102	SH214267.07FU	*Meliniomyces*	Endophyte				*	*
OTU53	SH181102.07FU	*Rhizoscyphus*	Endophyte					*
OTU502	SH203693.07FU	*Cadophora*	Endophyte		*	*	*	
OTU531	SH201720.07FU	*Leptodontidium*	Endophyte		*	*		
OTU5829	SH181085.07FU	*Meliniomyces*	Endophyte			*	*	*
OTU428	SH181102.07FU	*Rhizoscyphus*	Endophyte		*	*	*	*
OTU34	SH204990.07FU	*Phialocephala*	Endophyte					*
OTU101	SH214267.07FU	*Meliniomyces*	Endophyte				*	*
OTU299	SH214272.07FU	*Meliniomyces*	Endophyte			*	*	*
OTU138	SH275913.07FU	*Acephala*	Endophyte				*	*
OTU6855	SH029393.07FU	*Oidiodendron*	ErM			*		
OTU70	SH181078.07FU	*Meliniomyces*	ErM		*	*	*	*
OTU554	SH216991.07FU	*Oidiodendron*	ErM			*	*	*
OTU3661	SH191149.07FU	*Verticillium*	Pathogen		*	*	*	*
OTU6998	SH495464.07FU	*Arachnopeziza*	Saprotroph	*	*		*	
OTU4567	SH025039.07FU	*Cryptodiscus*	Saprotroph			*		
OTU1748	SH220728.07FU	*Mycena*	Saprotroph					*
OTU19	SH176953.07FU	*Galerina*	Saprotroph				*	*
OTU569	SH180492.07FU	*Archaeorhizomyces*	Saprotroph			*		
OTU561	SH180493.07FU	*Archaeorhizomyces*	Saprotroph	*		*		
OTU1797	SH176939.07FU	*Galerina*	Saprotroph					*
OTU4373	SH193238.07FU	*Cladophialophora*	Saprotroph		*	*	*	*
OTU5290	SH491946.07FU	Fungi	Unknown		*			
OTU2345	SH469369.07FU	Fungi	Unknown	*				*
OTU5023	SH181579.07FU	Chaetosphaeria	Unknown		*	*	*	*
OTU5150	SH201644.07FU	Hyaloscyphaceae	Unknown			*	*	
OTU6953	SH204499.07FU	Rozellomycota	Unknown				*	*
OTU4639	SH217841.07FU	Herpotrichiellaceae	Unknown			*	*	*
OTU4972	SH215043.07FU	Lecanoromycetes	Unknown		*	*	*	*
OTU3976	SH495485.07FU	Fungi	Unknown					*
OTU4919	SH201649.07FU	Helotiales	Unknown					*
OTU5850	SH201636.07FU	Dermateaceae	Unknown		*		*	*
OTU4442	SH193235.07FU	Herpotrichiellaceae	Unknown		*	*	*	*
OTU4416	SH012608.07FU	Chaetothyriales	Unknown				*	*
OTU13	SH211718.07FU	Helotiales	Unknown					*
OTU4614	SH458421.07FU	Herpotrichiellaceae	Unknown				*	

*^1^Fungal ecophysiological categories: ectomycorrhizal (EcM), ericoid mycorrhizal (ErM). ^2^Asterisks refer to significant relationship between sampling time and the occurrence of fungal out. All OTUs recorded from less than four samples were excluded from the indicative species analysis.*

Contrary to time effect, we detected significant effect of microhabitat on composition of fungal communities associated with control plants. However, the effect of microhabitat was more significant and explained two times more variability of fungal communities associated with decomposing spruce seedling roots compared to the control plants (Table 2). We did not identified significant effect of microhabitat × sampling time interaction on composition of root associated fungal communities. *Post hoc* comparison test identified significant differences in composition of fungal communities among all studied forest microhabitats. Numerous fungal OTUs associated with decomposing spruce roots showed significant affinity to one or two of the studied microhabitats (Table 4). Saprotrophic fungi such as *Mycena* sp. (OTU1377; SH1542291.08FU), *Chalara* sp. (OTU5132; SH1522496.08FU), and *Gyoerffyella* sp. (OTU5036; SH1509517.08FU) were predominantly found on spruce roots decomposing on CWD, particularly in early stages of the decomposition process. On the contrary, fungi with endophytic life strategy were more frequent on decomposing spruce roots in the ericoid shrubs. Interestingly, we identified clear segregation of two closely related endophytic fungi *Phialocephala* sp. (OTU374; SH1647307.08FU) and *Acephala* sp. (OTU551; SH2102217.08FU), also known as dark septate endophytes (DSE), among the studied microhabitats. While *Phialocephala* sp. dominated on decomposing spruce roots in ericoid shrubs, *Acephala* sp. was predominantly found in the other two microhabitats.

**TABLE 4:** Indicative fungal OTUs associated with roots of decapitated *Picea abies* seedlings across forest microhabitats 2–15 months after decapitation, with classification into fungal species hypothesis on 98.5% similarity level.

Sampling time	OTU	SH	Taxonomy	Ecology^1^	Indicative for microhabitat^2^		
					Bare forest soil	Ericoid shrubs	Coarse woody debris
2	OTU3392	SH213927.07FU	*Tylospora*	EcM		*	
	OTU3394	SH213927.07FU	*Tylospora*	EcM		*	
	OTU200	SH212614.07FU	*Hygrophorus*	EcM	*	*	
	OTU3359	SH213927.07FU	*Tylospora*	EcM	*	*	
	OTU48	SH181078.07FU	*Meliniomyces*	ErM	*		*
	OTU510	SH181093.07FU	*Meliniomyces*	Endophyte		*	
	OTU1377	SH220720.07FU	*Mycena*	Saprotroph			*
	OTU5132	SH202706.07FU	*Chalara*	Saprotroph			*
	OTU5036	SH184432.07FU	*Gyoerffyella*	Saprotroph			*
	OTU4615	SH217841.07FU	Herpotrichiellaceae	Unknown	*	*	
4	OTU555	SH216991.07FU	*Oidiodendron*	ErM	*		*
	OTU535	SH216987.07FU	*Oidiodendron*	ErM	*		*
	OTU4715	SH203224.07FU	*Leptodontidium*	Endophyte		*	
	OTU532	SH214267.07FU	*Meliniomyces*	Endophyte	*	*	
	OTU510	SH181093.07FU	*Meliniomyces*	Endophyte		*	
	OTU1377	SH220720.07FU	*Mycena*	Saprotroph			*
	OTU5036	SH184432.07FU	*Gyoerffyella*	Saprotroph			*
	OTU412	SH020043.07FU	*Collophora*	Unknown		*	
	OTU4640	SH217841.07FU	Herpotrichiellaceae	Unknown		*	
	OTU4498	SH219622.07FU	Fungi	Unknown	*		*
10	OTU264	SH211718.07FU	Helotiales	Unknown	*		*
15	OTU374	SH214294.07FU	*Phialocephala*	Endophyte		*	
	OTU551	SH275913.07FU	*Acephala*	Endophyte	*		*

*^1^Fungal ecophysiological categories: ectomycorrhizal (EcM), ericoid mycorrhizal (ErM). ^2^Asterisks refer to significant relationship between forest microhabitats and the occurrence of fungal OTU. All OTUs recorded from less than four samples were excluded from the indicative species analysis.*

## Discussion

In our study, we identified differences in composition of fungal communities associated with decomposing roots of *P. abies* seedlings in three different forest microhabitats. To get unbiased results, we decided to study fungal communities developing on decomposing roots of decapitated spruce seedlings rather than to use common bags filled with collected root biomass (e.g., Otsing et al., 2018; Barel et al., 2019). Due to nylon mesh or other fabric surrounding the decomposing roots, the interconnectivity with soil (the source of potential root decomposers) is limited, which can bias development of the fungal communities. Even more importantly, the collection of roots for litterbags destroys the context of decomposition since the roots are taken from their place. Our approach solves the above problems, but as a trade-off, it makes determination of root mass loss practically impossible. To overcome this obstacle, we used lignin and cellulose concentrations in decomposing root tissues as a proxy of decomposition. Because less recalcitrant compounds, such as proteins, hemicelluloses, pectin, etc., are utilized first during decomposition (Šnajdr et al., 2011), we considered a significant increase in lignin and cellulose concentrations in decomposing root tissue compared to control (living spruce roots) as indicator of decomposition processes. Based on our results, it seems that significant decomposition processes of root tissue started between 2 and 4 months after the seedlings decapitation regardless of the studied microhabitat.

Since we were interested in only trend-indicative values of cellulose concentration during the decomposition process, we used a spectrophotometrical method of determination of lignin and cellulose concentrations. The method was primarily derived for lignin determination (Lange et al., 1995) and in this method, cellulose content can be then determined from the same samples as lignin after repeated washing of the pellets after lignin extractions (Loader et al., 1997), what makes the method quite inexpensive though less precise for determination of absolute values. Thus, it should be mentioned that while the values of the lignin concentrations were found to be comparable with other studies, e.g., Hobbie et al. (2010), the values of cellulose concentrations in our study were found to be lower (only 20% of dry mass) and should be regarded as only trend-indicative values for comparison among treatments in the present study.

### Succession of Fungal Communities on Decaying Spruce Roots

To determine natural temporal changes of composition of fungal communities, we also sampled living spruce seedlings besides the decapitated ones during each sampling time. Sampling of these control plants revealed no effect of the sampling time on the fungal communities. Previous research conducted on the same sampling site (Žifčáková et al., 2016, 2017) also documented no significant change in fungal community composition among seasons, although the seasonal transcriptional activity differed among taxa.

Regardless of a forest microhabitat, we observed a clear shift in relative abundances of different fungal ecophysiological categories during succession of fungal communities on decomposing roots. Decapitation of the spruce seedling associated with termination of photosynthetic assimilates and other organic compound transport to root and adjacent soil resulted in a gradual decrease in relative abundance of EcMF. Although this observation corresponds to previously reported changes in fungal communities after disruption of belowground C transport induced by tree girdling (Högberg et al., 2001; Kaiser et al., 2010), clearcutting (Kohout et al., 2018; Parladé et al., 2019), defoliation (Parker et al., 2017), or bark beetle outbreaks (Štursová et al., 2014; Pec et al., 2017), we can identify some interesting differences among the studies. Previously, Štursová et al. (2014) documented the decrease in relative abundance of EcMF in the soils of spruce forests from 41% to less than 10% during 1 year after the dieback of all mature trees following a bark beetle outbreak. Similarly, relative abundance of ectomycorrhizal fungi declined from 55 to less than 5% 10 months after forest clearcutting (Kohout et al., 2018). On the contrary, in our experiment, the relative abundance of EcMF decreased from 65% before spruce seedlings decapitation to 40% 10 months after seedlings decapitation. Compared to the above mentioned reports, our experimental design only affected selected spruce seedlings, not mature trees. Therefore, the forest stands retained the same primary productivity and the major transport pathway of photosynthetic assimilates to mycelial network of EcMF on the study site was not disrupted. Our results indicate that the EcMF can persist on roots of decapitated seedlings longer in case that the common mycelial network is maintained by adjacent photosynthetically active host plants. The longer persistence of EcMF on decomposing fine roots may slow the decomposition processes as previously indicated by Langley et al. (2006). If the persisting EcMF suppress the rate of root decomposition by competition for the substrate with saprotrophs (Fernandez and Kennedy, 2016) or utilize some of the less recalcitrant compounds of decomposing plant tissue remains an open question.

According to our analysis, it seems that EcMF differ in their ability to persist on decaying host plant roots. Interestingly, *Russula* sp. and *Cortinarius* sp. were able to survive or even increase their relative abundance after the spruce seedlings decapitation. High relative abundance of *Russula* spp. after substantial decline of photosynthetic compound production has already been reported after moth outbreak (Saravesi et al., 2015) or bark beetle outbreak (Štursová et al., 2014; Treu et al., 2014). *Russula* spp. were also among the very few EcMF able to maintain their relative abundances on decomposing roots as well as in adjacent soil for some time after forest stand clearcutting as opposed to other EcMF species, e.g., *Amanita* spp. that disappeared very soon (Kohout et al., 2018). The ability to persist in soil or on host plant roots after termination or significant decrease of C flow from the host plants is likely not caused by slower decomposition of *Russula*’s biomass, because its decomposition rate was comparable with numerous other tested EcMF (Brabcová et al., 2018). We rather hypothesize that *Russula*’s mycelial network radiating from roots of mature living spruce was potentially able to mobilize nutrients from decomposing plant biomass due to lignocellulose degrading enzymes (Štursová et al., 2012; Miyauchi et al., 2020). The ability to decompose organic matter was also ascribed to the second recalcitrant genus found in our study, *Cortinarius* (Bödeker et al., 2014). The ability of *Russula* spp. and *Cortinarius* spp. to persist on the decomposing roots may lead to direct competition for resources with saprotrophs and result in an overall decrease in decomposition rate (Fernandez and Kennedy, 2016).

As previously documented (Kohout et al., 2018; Gray and Kernaghan, 2019), the proportion of endophytic fungi in decaying spruce seedling roots increased. These communities were dominated by endophytic *Meliniomyces* spp. previously reported as common co-colonizers of spruce ectomycorrhizas (Vohník et al., 2013). Besides the endophytes, some ErMF also increased their relative abundance in decaying roots. Ericoid mycorrhizal fungi may also act as endophytic colonizers of roots of non-ericoid plants (Chambers et al., 2008; Kohout et al., 2013) and compared to EcMF, they generally feature greater enzymatic capabilities involved in decay of lignin and cellulose (Vohník et al., 2012; Martino et al., 2018). Therefore, we suggest that besides endophytic fungi, ErMF play a role in decomposition of spruce roots.

Interestingly, a number of fungi, with unknown ecology indicative for different sampling times increased during the root decomposition, reached a maximum at the last sampling point. Although aboveground woody tissues in later stages of decomposition are usually colonized by brown-rot or white-rot fungi from Basidiomycota, these fungi were relatively scarce on decomposing *P. abies* tissues here as well as in a previous study (Kohout et al., 2018). The vast majority of the fungi with unknown ecology indicative for later stages of decomposition showed taxonomic affinity to Ascomycota. Clearly, decomposing roots represent so far overlooked niche for new fungal species that may play an important role in mineralization of belowground plant litter.

### Role of Microhabitat on Composition of Fungal Communities Associated With Decaying Spruce Roots

Although forest microhabitats differ in their biotic and abiotic conditions (St Hilaire and Leopold, 1995) and distinct EcMF communities were documented for the same host from different microhabitats (Iwański and Rudawska, 2007; Tedersoo et al., 2008), we found only small differences of fungal communities associated with living spruce seedling roots among microhabitats in a temperate mountainous spruce forest. Similarly, little effect of different microhabitats on EcMF communities was documented in high elevation spruce forest (Walker and Jones, 2013). Lack of the microhabitat effect on the composition of root associated fungal communities indicates that the spruce seedlings may tend to actively select similar fungal consortia in different forest microhabitats under some circumstances.

On the contrary, fungal communities on decaying spruce roots were more strongly structured by microhabitats, compared to fungal communities associated with living spruce roots. Effect of environmental conditions on decomposition rate as well as composition of fungal communities was previously documented for aboveground plant litter (Dang et al., 2009; Asplund et al., 2018) and fungal necromass (Maillard et al., 2020). Although our experimental approach did not allow us to determine root mass loss during the decomposition, we found substantial differences in composition of fungal communities associated with decaying roots among the studied microhabitats.

Although relative abundance of endophytic fungi in decaying roots increased across all studied microhabitats, we found a clear niche partitioning between *P. fortinii* s. l. and *A. applanata*, the two most well-known dark septate endophytes (Grünig et al., 2008). While *P. fortinii* s. l. associated preferentially with decomposing spruce seedling roots in the ericoid shrub context, *A. applanata* was almost completely missing in this microhabitat while it dominated on decomposing spruce roots in other two microhabitats. *A. applanata* was previously almost exclusively isolated from *P. abies* roots, whereas *P. fortinii* s. l. showed preference for roots of ericoid plants, but occurred also on spruce roots (Grünig et al., 2008). Therefore, ericoid plants may act as refuge for *P. fortinii* s. l. which may preferentially colonize roots spruce roots compare to *A. applanata*.

As hypothesized, we identified saprotrophic fungi, namely, *Mycena* sp., to be indicative for spruce roots decomposing on CWD, although the relative increase of *Mycena* sp. during the sampling time was observed in other microhabitats as well. Similarly, significantly greater relative abundance of saprotrophic fungi was recently documented on fungal necromass decomposing in course woody debris microhabitat than in bare forest soil (Maillard et al., 2020). *Mycena* spp. are known to be white-rot decomposers and they were also previously identified as dominant component of fungal communities in later stages of decomposing roots (Kohout et al., 2018) or deadwood (Baldrian et al., 2016). Considering that *Mycena* spp. possess greater enzymatic capabilities to degrade components of plant cell wall such as cellulose or lignin compared to EcMF or endophytic fungi (Miyauchi et al., 2020), our results indicate that its higher relative abundance in CWD microhabitat can stimulate decomposition of the root biomass.

On the contrary to our hypothesis, ErMF showed higher affinity to spruce roots decomposing in bare soil and CWD microhabitats. Ericoid mycorrhizal fungi possess the ability to form endophytic association with non-ericoid mycorrhizal plants (Chambers et al., 2008; Kohout et al., 2013). Therefore, the ericoid plant shrubs do not represent the only suitable habitat for ErMF. Why some ErMF preferentially colonize decomposing spruce roots remains an open question.

To conclude, our results indicate that the ectomycorrhizal fungi can persist on decaying roots of spruce seedlings longer in case that the common mycelial network is maintained by adjacent photosynthetically active host plants. We also identified stronger effect of forest microhabitat on structuring fungal communities associated with decaying compare to living roots of spruce seedlings. The difference was particularly expressed in endophytic fungi. Some ericoid mycorrhizal fungi showed higher relative abundance on decaying roots in soils under ericoid shrub cover, while saprotrophic fungi had higher relative abundance in roots decomposing inside deadwood.

## Data Availability Statement

The obtained data were deposited in NCBI SRA (PRJNA634919).

## Author Contributions

PK, PB, and JA jointly conceived the study. PK performed the field experiment. PK, RS, and SV analyzed the data. PK drafted the manuscript along with RS. VB and PB provided the chief contributions. All authors wrote and reviewed the manuscript.

## Conflict of Interest

The authors declare that the research was conducted in the absence of any commercial or financial relationships that could be construed as a potential conflict of interest.

## Footnotes

1 www.indexfungorum.org

## Supplementary Materials

Supplementary Figure 1 Concentrations of two plant structural biopolymers lignin **(A)** and cellulose **(B)** in root dry mass of living *Picea abies* seedlings (control plants) in the three studied microhabitats. Bars indicate means (*n* = 8) and whiskers represent standard errors. Different letters indicate significant differences among different sampling times within the same microhabitat.

Supplementary Table 2 List of OTUs detected in the study, with their corresponding number of reads, best BLAST match according to the UNITE database (ver. 7.1, release 2018-08-22), taxonomic affiliation (genus level) and ecology according to the FINGuild (“-” = unassigned). The final taxonomic and ecological classification has been manually corrected to our best knowledge when needed.

Supplementary Table 2 Fungal community matrix (read numbers of fungal OTUs in each sample in a dataset downsampled to a median of 16,024 reads). Each row represents an OTU, each column represents a sample. T0 = control sampling before decapitation, T1, T2, T3, and T4 = samplings at 2, 4, 10, and 15 months after decapitation, respectively, C, control spruce seedlings sampled 2–15 months after decapitation; B, bare forest soil; W, coase woody debris; Z, ericoid shrubs.
