# Supplementary figures and images for: Forest Microhabitat Affects Succession of Fungal Communities on Decomposing Fine Tree Roots
Source: Front Microbiol. 2021 Jan 28;12:541583. doi: 10.3389/fmicb.2021.541583 (PMC7876299; doi:10.3389/fmicb.2021.541583)

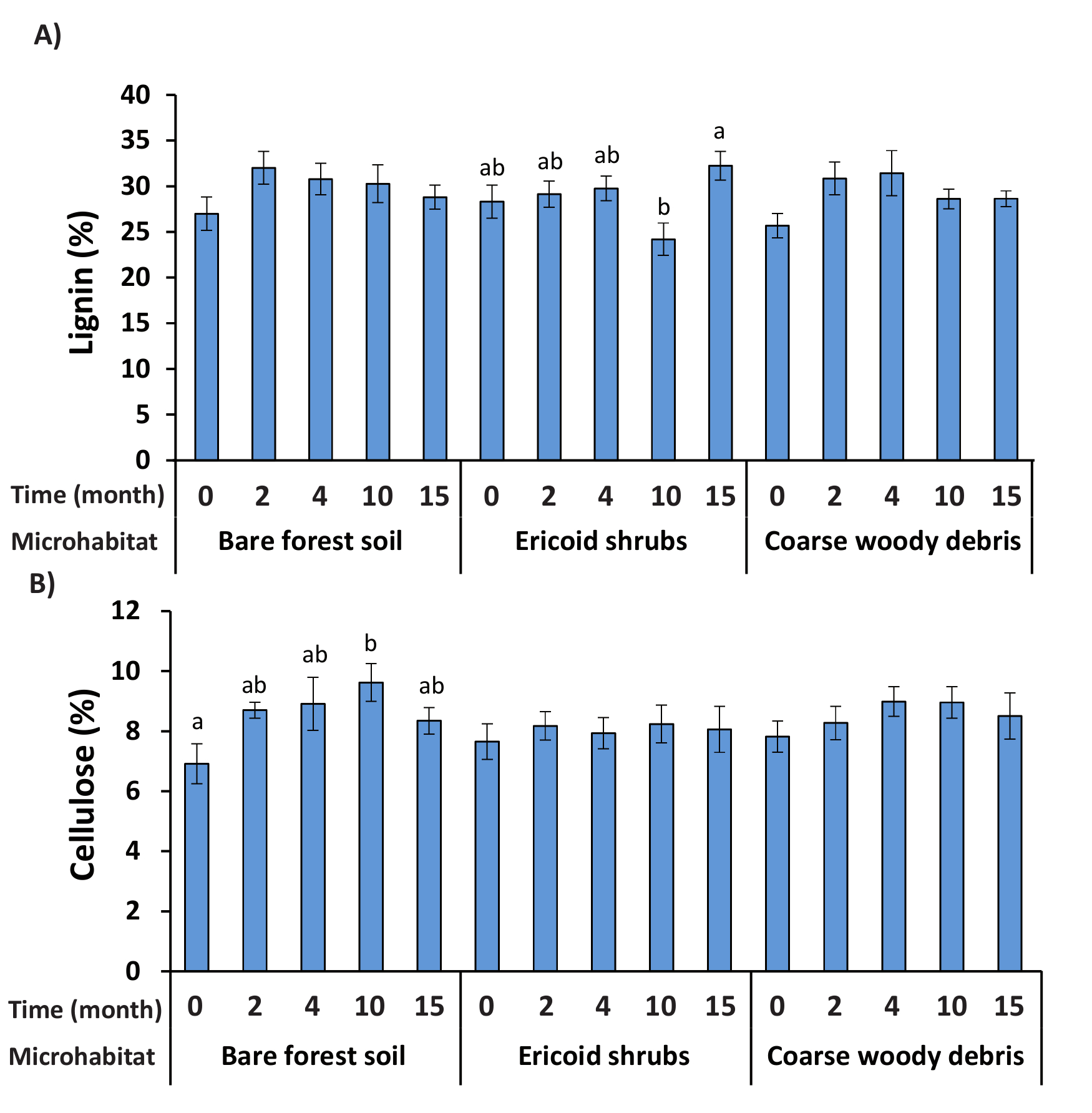

Supplement: Supplementary Figure 1 — Concentrations of two plant structural biopolymers lignin (A) and cellulose (B) in root dry mass of living Picea abies seedlings (control plants) in the three studied microhabitats. Bars indicate means (n = 8) and whiskers represent standard errors. Different letters indicate significant differences among different sampling times within the same microhabitat. [file Image_1.JPEG]
